# Time Changes with the Embodiment of Another’s Body Posture

**DOI:** 10.1371/journal.pone.0019818

**Published:** 2011-05-27

**Authors:** Francisco C. Nather, José L. O. Bueno, Emmanuel Bigand, Sylvie Droit-Volet

**Affiliations:** 1 University of São Paulo, Ribeirão Preto, Brazil; 2 Institut Universitaire de France, Université de Bourgogne, LEAD CNRS, Dijon, France; 3 University Blaise Pascal, LAPSCO CNRS, Clermont Ferrand, France; Duke University, United States of America

## Abstract

The aim of the present study was to investigate whether the perception of presentation durations of pictures of different body postures was distorted as function of the embodied movement that originally produced these postures. Participants were presented with two pictures, one with a low-arousal body posture judged to require no movement and the other with a high-arousal body posture judged to require considerable movement. In a temporal bisection task with two ranges of standard durations (0.4/1.6 s and 2/8 s), the participants had to judge whether the presentation duration of each of the pictures was more similar to the short or to the long standard duration. The results showed that the duration was judged longer for the posture requiring more movement than for the posture requiring less movement. However the magnitude of this overestimation was relatively greater for the range of short durations than for that of longer durations. Further analyses suggest that this lengthening effect was mediated by an arousal effect of limited duration on the speed of the internal clock system.

## Introduction

We are witnessing a renewal of interest in time distortions in human beings which suggest that judgments of time are affected by non-temporal dimensions [1], [2], [3]. Brown [4] examined the difference in the time judgments made by human adults when confronted with a stationary and a moving visual display composed of different geometric shapes. While the number of shapes did not affect time perception, the duration of the moving display was systematically judged longer than that of the stationary display. Furthermore, this lengthening effect increased with the speed of motion, with the duration being judged longer when the shapes moved quickly than when they moved slowly. Recently, Kaneto and Murakami [5] replicated these results by showing that a moving object was perceived to last longer than a static object. The judgment of time is thus intrinsically related to movements in space. The aim of the present study was to examine whether the perception of a static image of a body posture whose production has required more or less movement also affects time perception.

Using the temporal bisection task currently employed in the field of time perception in both animals and human beings [6], [7], [8], [9], [10], Chambon, Droit-Volet and Niedenthal [11] revealed that the presentation duration of faces of elderly individuals was underestimated compared to that of faces of young individuals. They explained their results within the theoretical framework of embodiment by suggesting that the participants embodied the slow movements of elderly people. This would therefore have slowed down the speed of their internal clocks. As suggested by the internal clock models [12], [13], when the internal clock runs more slowly, fewer time units (pulses, oscillations) are accumulated and time is judged shorter.

Nather and Bueno [14], [15] used pictures of objects representing body postures of dancers and pictures of sculptures of ballet dancers made by the impressionist artist Edgar Degas which represent a meticulous study of human bodies in motion [16], [17], [18]. In Nather and Bueno’s studies, participants were asked to rate each dancer picture on a subjective 7-point scale from “motionless” (1) to “moving” (7). The participants were also required to estimate the presentation duration (36 s) of these pictures in a reproduction task. The results indicated that the reproduced duration changed as a function of the amount of movement suggested by the static pictures. For example, the durations associated with the Degas sculptures were underestimated for the body postures involving little movement and overestimated for those involving a great deal of movement, and were judged accurately at the mid-point of the movement scale.

The data from Nather and Bueno [14], [15], [19] and Chambon, Droit-Volet and Niedenthal [11] are consistent with the growing body of evidence indicating that there is a close relationship between perception and action [20], [21]. Several imagery studies have shown that observing another individual performing an action activates the same brain areas in the perceiver as those that are involved in the action [22], [23]. Neurons - referred to as *mirror neurons* – are thought to trigger in the observer of the action in the same way as if he or she were performing the observed action. As stated by Lacoboni and Mazziotta [24], the *mirror neurons* provide “a simple mechanism for understanding the actions of the other”. They thus play a fundamental role in the representation of other people's actions. The problem that still needs to be resolved is to know whether motor activities affect the perception of time, even though there is as yet no direct neurological evidence of any overlap or interaction between the neural mechanisms involved in time perception, on the one hand, and motor activities on the other [25].

Recent studies have suggested that individuals reenact the sensory-motor activities associated with an action not only when they observe exactly the same action but also when these actions are partially masked. Freedberg and Gallese [26] suggested that simply observing a *static pattern* which reflects the state resulting from an action induces a reenactment of the movement that made it possible to produce this action. Studies in humans have also shown that individuals represent the continuation of movements based on static images [27] as well as the possible spatial and temporal trajectories of such movements [28], [29]. Static images of human body postures thus automatically induce a mental simulation of associated movements and the corresponding sensory-motor characteristics. Consequently, in the present study, we may assume that the perception of pictures of body postures will automatically result in a temporal distortion of the corresponding presentation duration due to the properties of the reenacted movement.

In the present study, we therefore decided to further qualify the movement represented in the Degas dancer sculptures [15] in order to select two body postures which differed from one another very significantly in terms of movements. There is ample evidence that movement is dependent on a certain state of arousal which plays a fundamental role in preparing the body to act. We consequently also assessed the level of arousal induced in individuals by the perception of each picture of a body posture (see Method). Several studies using emotional pictures (pictures from the International Affective Pictures System (IAPS), emotional faces) have shown that participants overestimate high-arousal pictures compared to low-arousal pictures [30]. However, as discussed in more detail below, this arousal effect of emotional pictures on time perception has been shown to be limited to brief durations shorter than 2–3 s [31], [32]. The arousing effect of pictures is thus somewhat transient or short-lived. We therefore assumed that the period of perception of pictures of body postures which involve a high degree of movement would be judged longer than that of body posture which involve less movement. However, if this effect is mediated by arousal, this temporal overestimation should be greater for short durations (<2–3 s) than for long durations, since the arousal effect triggered by the perception of pictures is transient.

In the present study, the participants therefore performed a temporal bisection task with two different duration ranges: a short (0.4/1.6 s) and a long range (2/8 s). In the temporal bisection task, the participants were initially presented with the short and the long anchor duration of each duration range displayed in the form of a square. In the test phase, they were then presented with these two anchor durations together with other intermediate durations which were presented in the form of two body posture pictures: one with less movement (“less-movement body posture”) and the other with more movement (“more-movement body posture”). The participant’s task was to judge whether each comparison duration was more similar to the short or to the long anchor duration.

## Results

**Figure 1:**
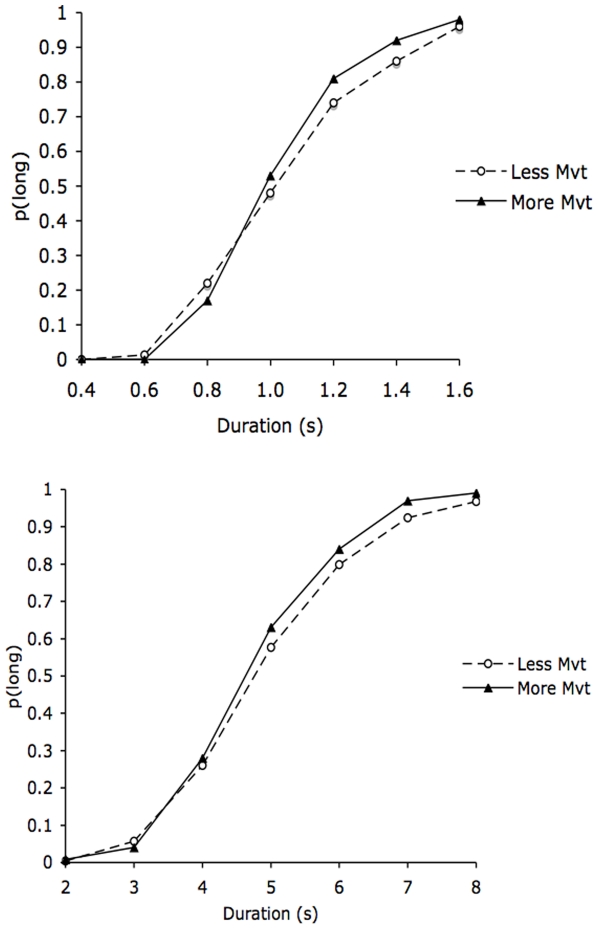
Psychometric functions for the body postures with more and less movements. Proportion of long responses plotted against the stimulus duration values for the body postures involving the production of a greater or lesser amount of movement for the short (0.4/1.6 s) and the longer (2/8 s) duration range.

Figure 1 indicates the proportion of long responses (*p(long)*) for the two body postures in the 0.4/1.6 s and the 2/8 s duration ranges. An examination of Figure 1 suggests that the bisection function was shifted toward the left for the body posture which involved more movement and was judged more arousing compared to the body posture involving less movement. This indicates that time was judged longer when the participants perceived a picture of a body posture which involved more movement. However, the magnitude of the leftward shift was relatively larger for the short than for the long duration range.

An ANOVA was run on *p(long)* with two within-subjects factors (body posture and comparison duration) and one between-subjects factor (duration range). The Greenhouse-Geisser Correction was used in this ANOVA as in the subsequent analyses. The ANOVA revealed no significant main effect of duration range, *F*(1, 48)  = 3.21, *p*>.05, or any significant interaction involving the duration range (all *p*>.05). In contrast, there was a significant effect of comparison duration, *F*(6, 288)  = 713.58, *p*<.05, and a significant interaction between comparison duration and body posture, *F*(6, 288)  = 2.27, *p*<.05, which subsumed no significant main effect of body posture, *F*(1, 48)  = 3.12, *p*>.05. This significant interaction indicated that the difference in *p*(long) between the body postures changed as a function of the comparison durations. As predicted, in the 0.4/1.6-s condition, the interaction between the body posture and the comparison duration reached significance, *F*(6, 144)  = 2.72, *p*<.05, with a significant main effect of comparison durations, *F*(6, 144)  = 343.69, *p*<.05, and no main effect of body posture, *F*(1, 24)  = 2.89, *p*>.05. According to the internal clock models [13], a temporal overestimation may be produced when the internal clock system runs faster (clock speed hypothesis) or when the attention-controlled switch which connects the pacemaker to the accumulator closes earlier (switch hypotheses). When the switch closure latency is shorter, more pulses are accumulated and time is judged longer. The mathematics underpinning the internal clock models nevertheless indicate that these two effects can be differentiated [33], [34]. The first of these models (clock speed hypothesis) should result in a multiplicative effect which would be larger for long than for shorter stimulus durations. The second (switch hypothesis) yields an additive effect which is constant across different stimulus durations. Consequently, in the 0.4/1.6-s duration condition, we calculated the difference in *p(long)* between the more- and less-movement body postures for the shorter (mean of the 3 shortest comparison durations) and the longer comparison durations (mean of the 3 longest comparison durations). The one-sample *t* test revealed that the magnitude of the differences between body postures was significantly greater than zero for both the short, *t*(24)  = 2.07, *p* = .05, and the long comparison durations, *t*(24)  = 2.92, *p* = .01). In addition, the magnitude of this between-body posture difference was larger for the long than for the shorter comparison durations (*t*(24)  = 3.61, *p* = .001). This suggests that there is a multiple effect, a finding which appears to be consistent with the clock rate hypothesis. In addition, we also performed statistical analyses on the y-intercept and the slope index of the individual psychometric functions, since the finding of an effect on the y-intercept or on the slope index would provide support for the switch mechanism and clock rate mechanism hypotheses, respectively [33], [34], [35]. These 2 measures were obtained by fitting the logarithmic function to each subject’s data. The logarithmic fit was significant for all the subjects, with a mean R^2^ of .86 (*SD* = .06). The body posture effect was significant for the slope index, *F*(1, 24)  = 4.53, *p*<.05, but not for the intercept, *F*(1, 24)  = .896, *p*>.05). Overall, our results therefore suggest that the overestimation of time for the more-movement body posture compared to the less-movement body posture is due to a clock rate effect, with the clock running faster for the body posture whose production requires more movement.

According to the internal clock models, if viewing a body posture which is associated with more movement affects the clock rate, this clock-related effect should be greater for longer than for shorter duration ranges. However, as we suggest in the introduction, the perception of arousing pictures is complex in that it produces a transient change in individuals’ states for up to 2–3 s [31], [32]. In line with this idea, although the omnibus ANOVA found a general body posture effect on *p*(long) irrespective of the duration range, the ANOVA for the long duration range (2/8 s) taken separately did not indicate any effect of body posture or any body posture × comparison duration interaction (*F*(1, 24)  = 1.07, *F*(6, 144)  = .62, respectively, all *p*>.05). There was only a significant main effect of comparison durations, thus indicating that *p*(long) increased with the value of the stimulus durations, *F*(6, 144)  = 372.02, *p*<.05.

Two other timing measures used to take account of temporal performance in bisection were also calculated, i.e., the bisection point (BP) and the Weber Ratio (WR). The BP is the point of subjective equality, i.e. the comparison duration (*D*) equally judged long and short: *D*(*p*(long))  = .50. The WR is the Difference Limen (*D*(*p*(long)  = .75) - *D*(*p*(long)  = .25)/2) divided by the BP. The Weber ratio is a sort of coefficient of variation. When Weber's law holds, the WR remains constant across different duration values. These 2 measures were derived from the slope and intercept parameters obtained from the significant fitting of a logarithmic function to the individual subject data (with the logarithmic functions, the WR values appeared relatively high). Table 1 summarizes the group means for the BP and WR in each experimental condition.

**Table 1:** Bisection Point and Weber Ratio for the body postures in the 0.4/1.6 and the 2/8-s duration condition.

	BP		WR	
	M.	*S.E.M*	M.	*S.E.M*
**0.4/1.6-s**				
Less-body posture	0.98	*0.04*	0.32	*0.01*
More-body posture	0.91	*0.03*	0.32	*0.01*
**2/8-s**				
Less-body posture	4.65	*0.14*	0.32	*0.02*
More-body posture	4.40	*0.11*	0.29	*0.01*

M.  =  Mean; S.E.M.  =  Standard Error of Mean; BP  =  Bisection Point; WR  =  Weber Ratio.

The ANOVA run on the BP with body posture duration range as a factor found a main effect of duration range, *F*(1, 48)  = 1023.54, *p*<.05, thus indicating that the BP was higher in the 2/8-s than in the 0.4/1.6-s duration condition. More interestingly, the effect of body posture reached significance, *F*(1, 48)  = 4.87, *p*<.05, while the duration × body posture interaction was not significant, *F*(1, 48)  = 1.60, *p*>.05. However, the ANOVA for each duration range taken separately showed that there was a significant body posture effect in the 0.4/1.6-s duration condition, *F*(1, 24)  = 4.16, *p*<.05, while this effect just failed to reach significance in the 2/8-s duration condition, *F*(1, 25)  = 3.20, *p*>.05. A significantly lower BP value confirmed that the comparison durations were judged longer for the more-movement than for the less-movement body postures. This finding, however, applied to short rather than longer durations (>2 s).

**Figure 2:**
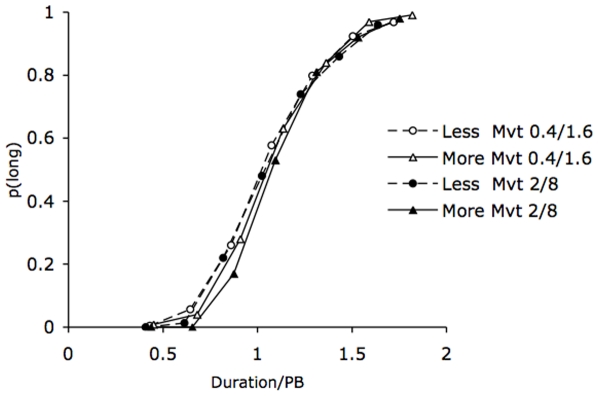
Superimposition between psychometric functions for the body postures. Proportion of long responses plotted against the stimulus duration values divided by the bisection point for the body postures involving the production of a greater or lesser amount of movement for the short (0.4/1.6 s) and the longer (2/8 s) duration range.

The ANOVA run on the WRs revealed no effect of duration, *F*(1, 48)  = 2.39, *p*>.05, or of body posture, *F*(1, 48)  = 2.51, *p*>.05, or any body posture × duration interaction, *F*(1, 48)  = 1.67, *p*>.05. In both the 0.4/1.6 and the 2/8-s duration conditions, the effect of body posture was not significant (*F*(1, 24)  = .06, *F*(1, 24)  = 3.30, respectively, all *p*>.05). This confirms that, at least for the short duration range, the difference between body postures was, in proportional terms, consistent with a clock rate mechanism that would have produced a proportional (multiplicative) rather than an absolute effect (additive). Our results also indicate that the scalar property holds, with a constant WR value being observed across different duration values. To test this scalar property, we also verified whether the psychometric functions superimposed well when they were normalized by the comparison duration divided by the BP. Figure 2 indicates a good superimposition between the psychometric functions obtained with the different body posture pictures, and especially in the short duration condition. In the case of the long duration condition, there was a slight rightward shift of the psychometric function for the more-movement body posture. Post-hoc comparisons revealed that the WR was significantly lower in the 2/8 than in the 0.4/1.6-s condition in the case of the more-movement body posture, *t*(48)  = 2.51, *p*<.05, whereas the WR was similar in the two duration range conditions in the case of less-movement body postures, *t*(48)  = .37, *p*>.05. Consequently, the participants tended to be less variable in the timing of long durations when they were presented with the more-movement body posture.

## Discussion

While the results of the present study reveal that the perception of the duration of pictures depicting body postures changes as a function of the movement implied by this posture, they also show that this is true of duration ranges shorter rather than longer than 2 s. Indeed, for the short duration range (0.4/1.6-s), the bisection function was significantly shifted toward the left, with a lower BP for the posture whose production required a large movement than for the posture requiring no movement. In other words, the duration was judged longer for the more-movement than for the less-movement body posture. This result is consistent with those found by Brown [4] and Kaneto and Murakami [5] which showed that the duration of a moving display is judged longer than that of a static display. However, the originality of our study is to show, using a bisection task, that the time lengthening effect also occurs with static images of body posture which induce a perception of movement [14], [15].

The participants' assessment of the pictures used in the present study revealed that the body posture involving more movement was judged more arousing than that involving less movement. By manipulating a great variety of arousing conditions (drugs, emotion, click train), a large number of studies have shown that when the nervous system is aroused, the clock-like system speeds up and more time units (pulses) are accumulated, with the results that the elapsed duration is judged longer [8], [9], [36], [37]. In line with this finding, we can assume that the temporal overestimation of the body posture requiring more movement was due to the increase of the clock rate which was, in turn, mediated by an increase in the level of arousal produced by the perception of this posture. Some of the evidence derived from our data supports this hypothesis. First, the between-body posture difference increased with the value of the comparison durations and a significant slope effect was also observed. Second, this difference was proportional, with a constant WR and a good superposition being observed between the psychometric functions associated with the body posture. The question that remains to be answered relates more to the causes of this speeding up of the internal clock. According to the internal clock models [13], a slope effect in the psychometric functions could be produced either by the acceleration of the pacemaker or by the flickering of an accumulator-switch system [33], [35], [38], [39]. In this latter case, it would be easier for the switch to remain closed during the processing of time in the case of the more-movement than the less-movement body posture. However, if this is this case, it is unclear why the body posture effect decreased for the longer duration range in our study.

The internal clock models predict that the clock-related effect (pacemaker or accumulator/switch system) should be larger for long durations than for short durations. Although we found an increase in the magnitude of body posture-related differences with the comparison durations in the 0.4/1.6-s condition, we did not find any effect in the longer duration conditions (2/8 s). The core problem lies in the dynamic of the individual states produced by the perception of arousing pictures. Using pictures from the IAPS that were rated as highly arousing on the basis of physiological indexes (heart rate, skin conductance), Angrilli et al. [31] observed a significant temporal overestimation of the presentation duration of high-arousal pictures compared to that of low-arousal pictures for the 2 s condition only. At the longer durations of 4 and 6 s, the high-arousal pictures were underestimated rather than overestimated. The authors explain their results in terms of two different mechanisms which emerge as a function of the durations to be estimated: an arousal-based mechanism for brief durations and an attention-based mechanism for long durations. A time-related shift therefore occurs from an activation to an attention-based mechanism for the processing of emotional pictures. Similarly, Bar-Haim et al. [32] observed a significant overestimation of fearful faces compared to neutral faces at 2 s. This lengthening effect persisted to a small extent at 4 s but totally disappeared at 8 s, as if time perception returns to its baseline state. As these authors state, there is no reason why a threat should capture attention for an extended period since the biological process of activation is rapid. Our results are thus entirely consistent with the findings of these studies, and suggest that there is a transient arousal effect which is produced by the perception of the body posture which involves more movement. Indeed, there is no reason why the initial attention effect was not maintained at long durations of up to 8 s. Finally, the predictions of the internal clock models concerning the existence of larger arousal effects for long than for short durations do not apply to durations longer than 2–3 s whatever the experimental situations, namely in the context of the perception of high-arousal pictures on the contrary to that of drug administration [9]. According to Nather and Bueno [14], who employed a reproduction task with long durations (36 s), the visualization of static body postures activates distinct cognitive processes as a function of the passage of time, associated both with the decay of arousal effects and with the different cognitive strategies which emerge during time estimation tasks [40], [41], [42], [43].

Our results demonstrate that the presentation duration of pictures was overestimated in response to the perception of a body posture involving more movement. This may be explained by the partial reactivation of the motor states involved in the movement that led to this posture (muscle contraction, cortico-spinal activities, etc.). As reported in the introduction, neuroimaging studies of mirror motor systems in monkeys and humans have shown that the observation of body movements activates the brain areas involved in the performance of the action in the perceiver [44]. According to Fadiga, Craighero and Olivier [45], the observation of an action triggers a specific activation of the muscles involved in the planning and the implementation of this action. In addition, this has been demonstrated not only when the action is perceived in its entirety (a hand taking an object), but also when the action can be inferred from cues (a hand next to an object). Indeed, even when the real motion is not present but implicit, human beings are capable of both recognizing and anticipating the movement of visual stimuli [46], [47], [48]. As shown by Urgesi et al. [47], seeing a photograph of a hand holding something is enough to produce an increase in cortical-spinal excitability related to the observation of this hand. Thus, in our study, we may assume that the presentation duration of the body posture associated with a large movement was judged to last longer because it involved the embodied simulation of a more effortful and arousing movement. Further studies involving sensory-motor indices will nevertheless be required if we are to provide evidence in support of this assumption.

Time illusions are the subject of a growing number of studies which have revealed that duration distortions can be induced by the properties of the stimuli themselves [2]. Our results are consistent with this observation in that they showed that a body posture associated with a considerable movement was perceived to last longer, at least for duration ranges shorter than 2 s. The originality of our study was thus to show that this time distortion also occurred when the properties of the stimuli were not directly perceived but reactivated in memory [19]. Our study also provides data suggesting that the internal clock runs faster with the embodied simulation of movements associated with body postures perceived in another person. This time distortion in the perceiver may thus be an index of empathic processes which enable him or her to understand the other and time his or her own action as a function of the timing of action observed in the other [49]. In sum, our study suggests that the judgment of time seems to be grounded in sensory-motor and affective states experienced or reenacted in memory.

## Materials and Methods

### Participants

A total of fifty students (22 men and 28 women, mean age  = 21.90, *S.D.* = 3.73) from São Paulo University took part in this experiment. All participants gave written consent in accordance with the procedure approved by the ethics committee of the College of Philosophy, Sciences and Letters of Ribeirão Preto, University of São Paulo (USP), Brazil.

### Stimuli

**Figure 3:**
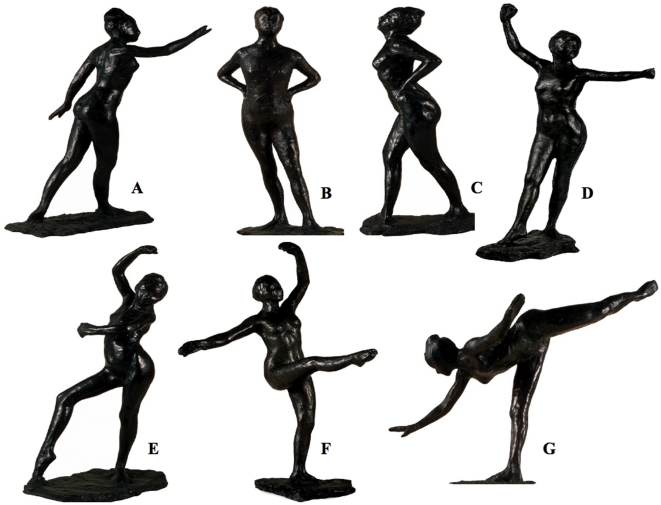
The 7 sculptures by Edgar Degas assessed in the present study. These 7 sculptures represent different body positions (ballet steps) and suggest movements of distinct intensities: (A) First movement of the great arabesque, (B) Ballerina at rest with her hands on her waist and her left leg in front (facing forward), (C) Ballerina at rest with her hands on her waist and her right leg in front (facing to the right) (D) Prelude to dance, with her right leg in front, (E) Spanish Dance, (F) Fourth position in front on the left leg, (G) Third movement of the great arabesque. *(Edgar Degas. Paris, France 1834-1917, MASP Collection, Museum of Art of São Paulo Assis Chateaubriand. João L. Musa’s pictures).*

The participants were seated in a laboratory room in front a *19"* screen connected to a PC. *E-Prime* software was used to control the experimental events and record the responses. The participants gave their responses by pressing one of two keys (D and K) on the computer keyboard. The stimuli to be timed consisted of a black square (10 cm) and the pictures of two sculptures of dancers by Edgar Degas depicting body positions which involved different movements, i.e., sculpture B and sculpture G (Figure 3). Photoshop was used to standardize the pictures of these sculptures, with the result that they were of the same size (30×40 cm) and quality (color saturation, brightness, contrast and resolution). The pictures were presented in the center of the computer screen.

These two sculptures were selected from a set of 7 different sculptures by Edgar Degas (Figure 3) which had been pre-tested in a sample of 25 additional participants (12 men and 13 women, mean age  = 20.96, *S.D.* = 1.51). These participants saw the sculpture pictures presented in a random order and were asked to observe the movement suggested by each body position and rate, on a 5-point scale, the amount of action required to perform it. They were also asked to rate their level of arousal using the Self-Assessment Manikin scale (SAM) [50]. A principal component analysis (PCA) was conducted on the mean data with the 7 sculptures as samples and the scales as variables. This analysis revealed that, on the major vector that explained 85% of variance, sculpture B and sculpture G were at the opposite ends of the scale, while the other sculptures occupied intermediate positions. Sculpture G was thus judged both to require more action and to be more arousing. The *t*-tests confirmed that the movement associated with sculpture G was judged to be more arousing (3.93 vs. 1.32, *t*(24)  = 10.61, *p* = .001), and more action-intensive (4.71 vs. 1.20, *t*(24)  = 10.55, *p* = .0001) than the movement associated with sculpture B. The participants therefore clearly differentiated between sculptures G and B on the basis of the movement involved.

### Procedure

The participants were assigned to two duration groups. In the 0.4/1.6-s duration group, the standard durations were 0.4 and 1.6 s, and the comparison durations 0.4, 0.6, 0.8, 1.0, 1.2, 1.4 and 1.6 s. In the 2/8-s group, the standard durations were 2 and 8 s and the comparison durations 2, 3, 4, 5, 6, 7 and 8 s. In each duration group, the bisection task consisted of a training phase and a test phase. In the training phase, the participants performed 6 trials with the short and the long anchor durations presented 3 times each in the form of a black square. In the experiment reported here, no feedback was given since it was easy for the adults to differentiate these two durations [10]. The trial order was random and the inter-trial interval was randomly chosen between 1 and 3 s. The participants were trained to press one key after the short standard duration and the other key after the long standard duration, with the button press assignment being counterbalanced. The same procedure was used in the test phase, except for the fact that the durations were presented in the form of the pictures of the two sculptures exhibiting different body postures: “*more movement*” (sculpture G) vs. “*less movement”* (sculpture B). The participants were instructed to press one key when they judged the comparison duration to be more similar to the short than to the long standard and the other key when they judged it to be more similar to the long than to the short standard. The participants were presented with 9 blocks of 14 trials each, i.e. 7 comparison durations × 2 body positions. Within each block, the trials were presented randomly. In addition, the experimenter clearly informed the participants of the importance of not counting and told them that counting would distort the results.
